# Investigation of temperature change during interproximal reduction and subsequent polishing

**DOI:** 10.1186/s12903-026-08194-w

**Published:** 2026-03-30

**Authors:** Ece Basaran, Sabri Ilhan Ramoglu

**Affiliations:** Department of Orthodontics, Dentistry Faculty, Altinbas University, Zuhuratbaba, Incirli Cd. No:11, Bakirkoy, Istanbul, 34147 Turkey

**Keywords:** Interproximal reduction, Polishing, Temperature change

## Abstract

**Background:**

Interproximal reduction (IPR) is widely used in orthodontics, but the heat generated during this procedure may affect pulp tissue. The aim of this study is to evaluate the temperature changes in the pulp chamber of lower incisor teeth during IPR process and subsequent polishing, by simulating the pulpal microcirculation, with a thermal camera.

**Methods:**

Intact and extracted 21 lower incisors were prepared and placed on the pulpal microcirculation model were treated with 0.3 mm IPR with perforated diamond-coated disc. As second step, the samples were polished for 80 s with fine Sof-Lex discs. On one surface of the teeth, the polishing process was carried out dry, on the other surface with water cooling. The initial pulp chamber temperature (T_0_), 45th second (T_1_), 60th second (T_2_), and 80th second (T_3_) temperatures were recorded with a thermal camera.

**Results:**

The temperature change (∆T) at the IPR was measured as 2.74 ± 1.4 °C. ∆T were measured as 2.74 °C and 3.73 °C, respectively, at the water cooling and dry polishing groups. T_1_, T_2_, T_3_, T_max_ and ∆T temperature measurements of water cooling and dry polishing groups show statistically significant differences (*p* < 0.001). ∆T measurement of the dry polishing group was found higher, but temperature increase above the critical value of 5.5 °C was not detected in both groups.

**Conclusions:**

Statistically significant temperature increase occurs in both IPR and polishing application. As a result of the polishing with or without water cooling, no temperature increase above 5.5 °C occurred. The temperature increase recorded during the 80 s polishing with Sof-Lex discs following IPR was not sufficient enough to harm pulp tissue.

**Practical implications:**

Although a statistically significant temperature increase was observed during both IPR and polishing, no temperature increase above 5.5°C was detected during IPR. The higher temperature increase observed with dry polishing compared to water cooling suggests that water cooling is an effective method to reduce temperature increase. The temperature change during the 80 second polishing with Sof-Lex discs following IPR is not at a level that would harm the pulp tissue.

**Supplementary Information:**

The online version contains supplementary material available at 10.1186/s12903-026-08194-w.

Interproximal reduction (IPR), also known as stripping, refers to the intentional removal of dental enamel from the interproximal contact areas, reducing the mesiodistal (MD) width of a tooth without compromising its structural integrity [1]. This technique is commonly employed to alter tooth shape, enhance gingival contour, eliminate black triangles, and adjust the Curve of Spee, all within the enamel surface [2]. Additionally, IPR can be used to create space during orthodontic treatment or to correct tooth size discrepancies, aiming for improved occlusion and interdigitation [1]. 

This irreversible procedure necessitates thorough examination prior to treatment [3]. The long-term disadvantages of IPR are still not fully understood [1]. Improper execution of IPR may lead to hypersensitivity, irreversible damage to the dental pulp [4, 5], increased plaque accumulation, a higher susceptibility to caries [6–12], and periodontal disease [13] in the treated enamel areas. Studies indicate that the roughened enamel surface following IPR may enhance bacterial adhesion, though polishing can mitigate surface roughness [14, 15]. 

While some authors report that even using the finest finishing strips cannot completely eliminate furrows on the enamel surface [7, 10, 11, 16, 17], other studies have found that polished enamel surfaces can be smoother than untreated enamel [12, 18, 19]. It has been suggested that longer polishing times result in smoother surfaces [18, 19]. 

Another potential side effect of IPR is the heat generated during the procedure [4, 20]. Zach and Cohen reported that a temperature increase of more than 5.5 °C in the pulp can cause inflammation [21]. Techniques using rotary instruments have been shown to generate heat, which may harm the pulpal tissues if not properly cooled [4]. 

Studies measuring the temperature increase during polishing on various composite and amalgam restorative materials have observed that the rise in temperature can exceed critical levels [22–24]. However, no studies in the literature have specifically addressed the temperature increase associated with polishing following IPR on enamel, highlighting a gap in understanding its potential effects on pulpal health.

It has been argued that in the presence of external stimuli applied to the dentine-pulp complex, the simulation of vascular microcirculation of the pulp is one of the important factors that significantly influence temperature rise in the pulp chamber [25, 26]. Therefore, in studies where pulpal microcirculation is not simulated, it is possible to obtain higher and misleading values when evaluating the temperature increase in the pulp chamber [25, 27]. 

The null hypothesis of this study is, although a temperature rise may occur during IPR and subsequent polishing, it does not exceed the critical threshold capable of causing irreversible damage to the pulpal tissue. Therefore, the aim of this study is to evaluate the temperature rise in the pulp chamber caused by IPR followed by a polishing sequence and comparing different techniques, in an effort to make in vitro conditions more reflective of in vivo conditions by simulating pulpal microcirculation using a thermal camera, marking it as the first study of its kind in the literature.

## Materials and methods

In the present study, 39 lower incisor teeth extracted for orthodontic and periodontal reasons at Altınbaş University Dental Hospital were obtained. Ethics committee approval was received for our study from the Altınbaş University Clinical Research Ethics Committee (protocol no: 2021/98), and all subjects signed informed consent.

**Fig. 1:**
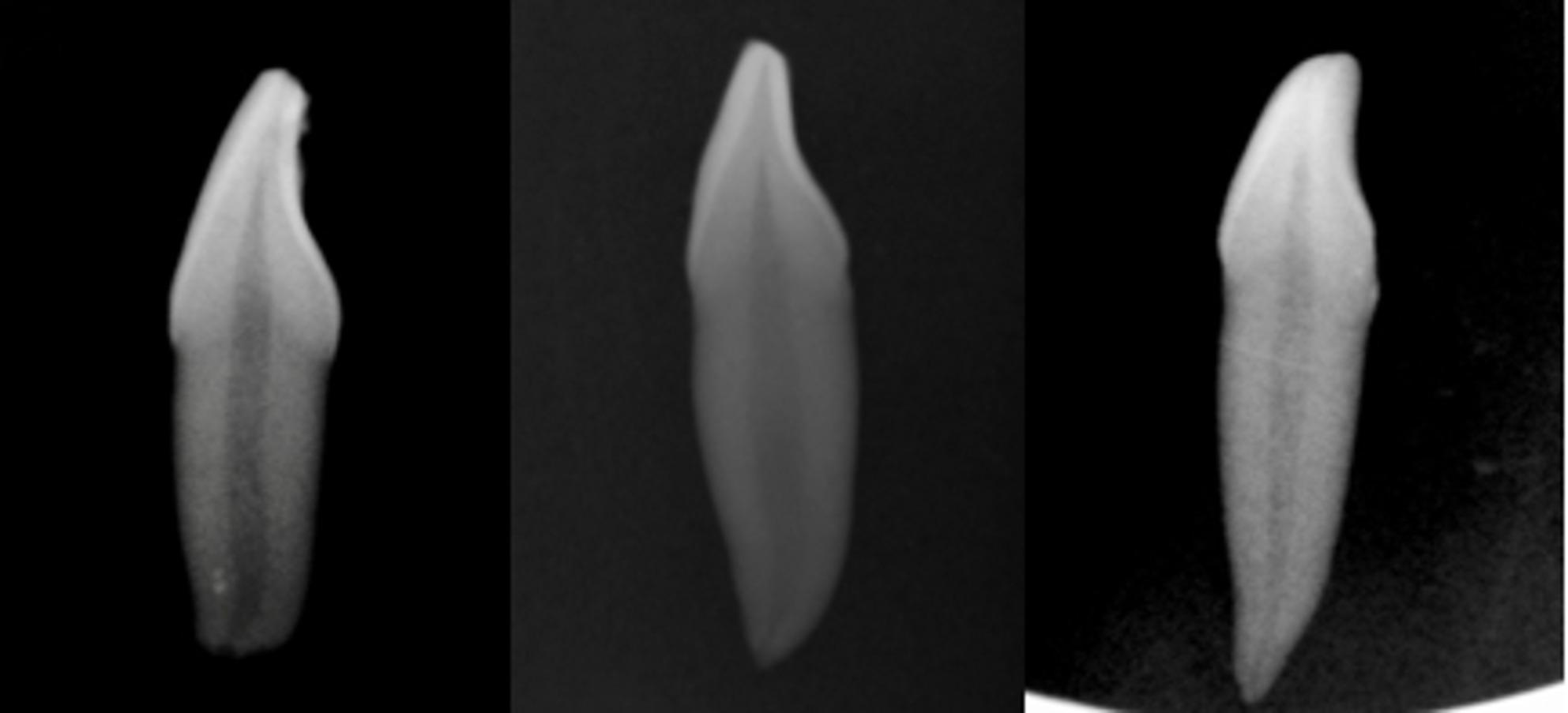
Periapical radiographs taken from extracted lower incisors

The total number of samples to be used in the present study was found by using the G-Power program and determined as *n* = 15, with an effect size of 0.90, 95% power, and a margin of error of 0.05, based on the values of the methods to be studied. As a result of inspection made on the 39 teeth, 11 teeth with caries, white spot lesions, changes in enamel structure and morphology, material loss on proximal surfaces, fluorosis, restorations, and enamel fractures were excluded from the study [5, 7, 11, 12, 18, 28, 29]. As a second step, examination made with periapical radiography taken from each tooth, 7 more teeth were excluded from the study, as it was determined that the pulp chamber was too narrow or wide (Fig. 1). It is aimed to ensure that the teeth and pulp chamber are homogeneous in size and shape [5, 30, 31]. Accordingly, the inclusion criteria were determined as teeth without caries, white spot lesions, changes in enamel structure, fluorosis, restoration, enamel fractures and the teeth which pulp chamber was not too narrow or wide. 21 teeth that met the inclusion criteria for the study were stored in distilled water at room temperature. Distilled water was changed weekly [26, 32, 33].

**Fig. 2:**
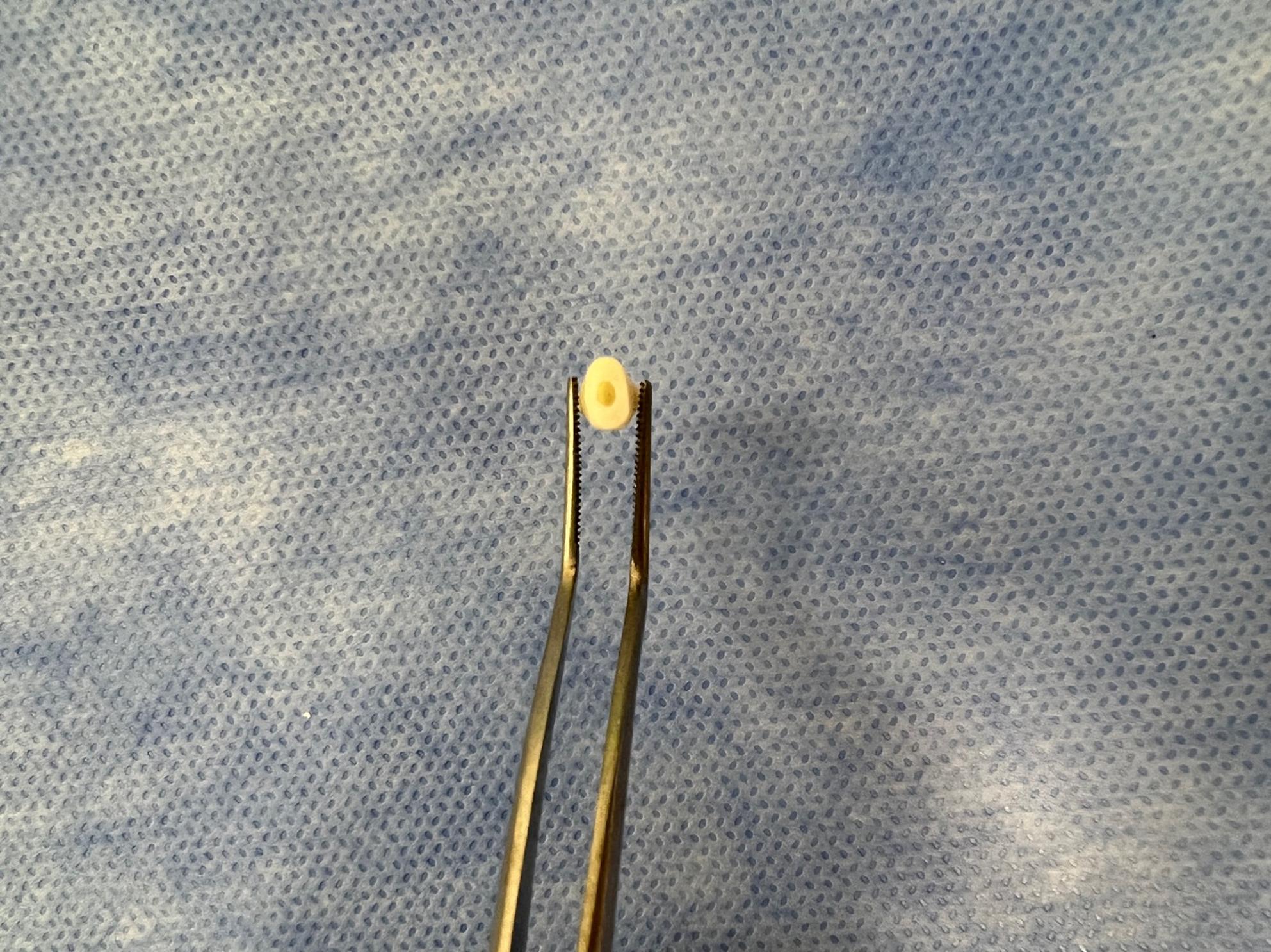
Tooth sample separated and prepared for study

In order to simulate pulpal microcirculation, plaque-free teeth were separated with a carbon disc from 2 mm apical to the cementum-enamel junction, perpendicular to the long axis of the teeth. The pulp chamber entrance was enlarged with a diamond bur, and the remaining pulp residues were cleaned with an excavator (Fig. 2). The pulp chamber was washed and air-dried [25, 26, 30, 32, 34–36].

**Fig. 3:**
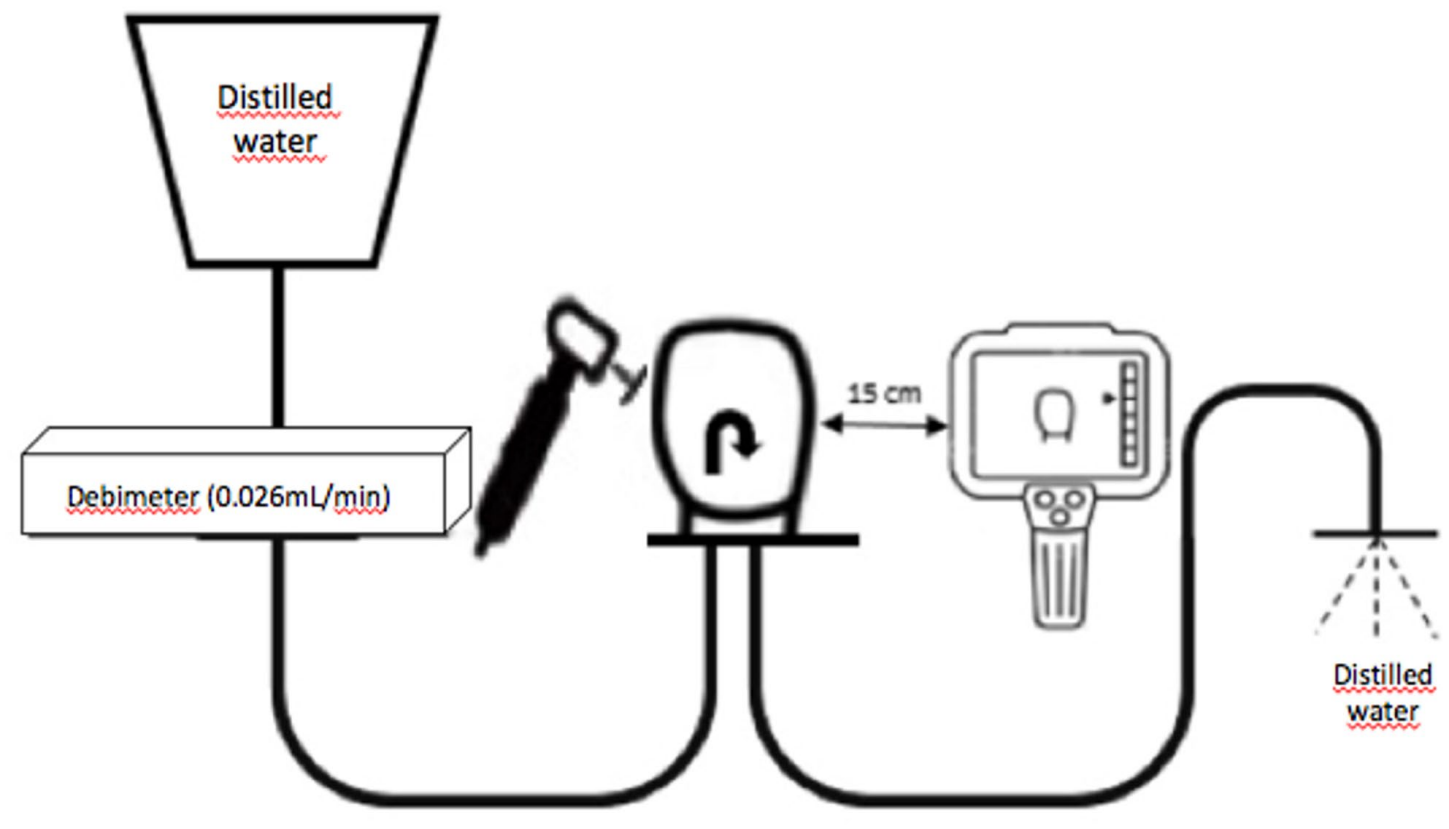
Schematic image of the pulpal microcirculation simulation model

The samples were placed on the mechanism where pulpal microcirculation was simulated, referencing Sari et al. [26]. With this setup, the flow of distilled water at room temperature was ensured through the sample by adjusting the flow rate of 0.026 mL/min, which is the vascular microcirculation rate of the pulp, with a flowmeter (SK-600II infusion pump, SK Medical, Shenzhen, China) [37]. The blood pressure of the pulp was adjusted to 15 mm H_2_O (Fig. 3) [26]. 

**Fig. 4:**
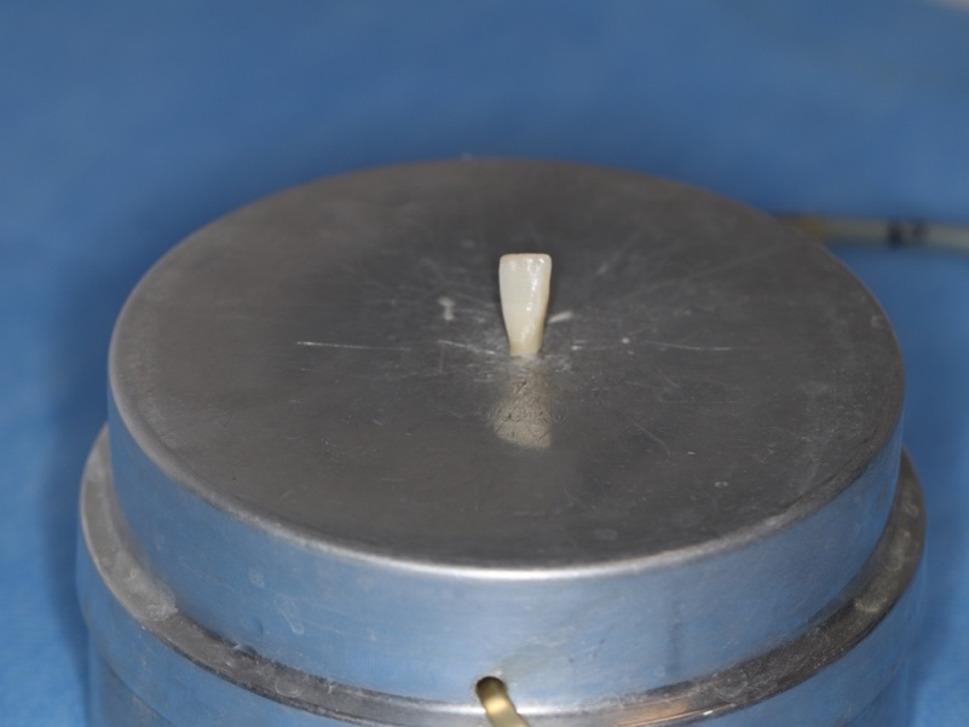
Sample placed on pulpal microcirculation model

**Fig. 5:**
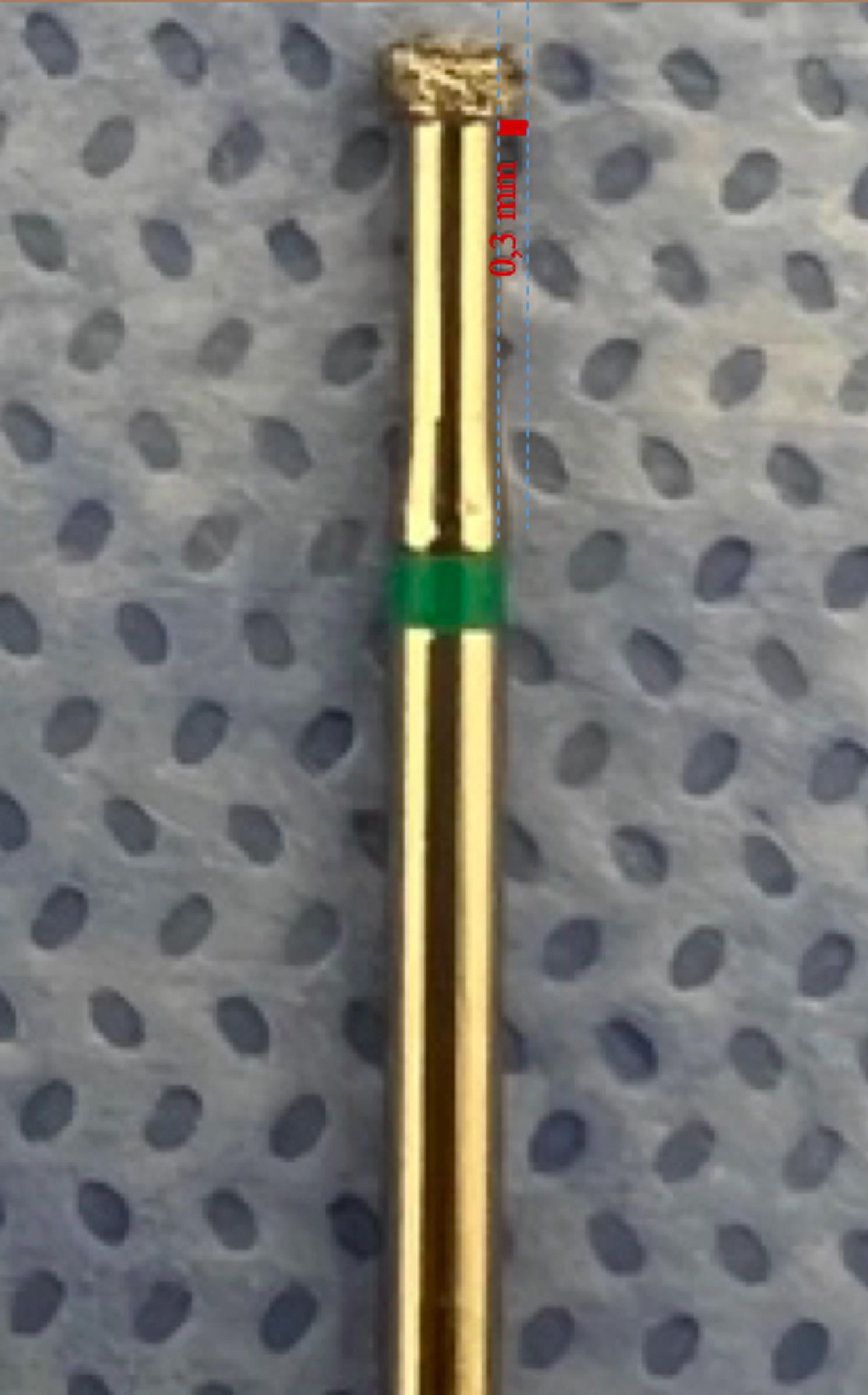
Bur used to standardize the amount of IPR applied (Intensiv, 818 102AC, Montagnola, Switzerland)

**Fig. 6:**
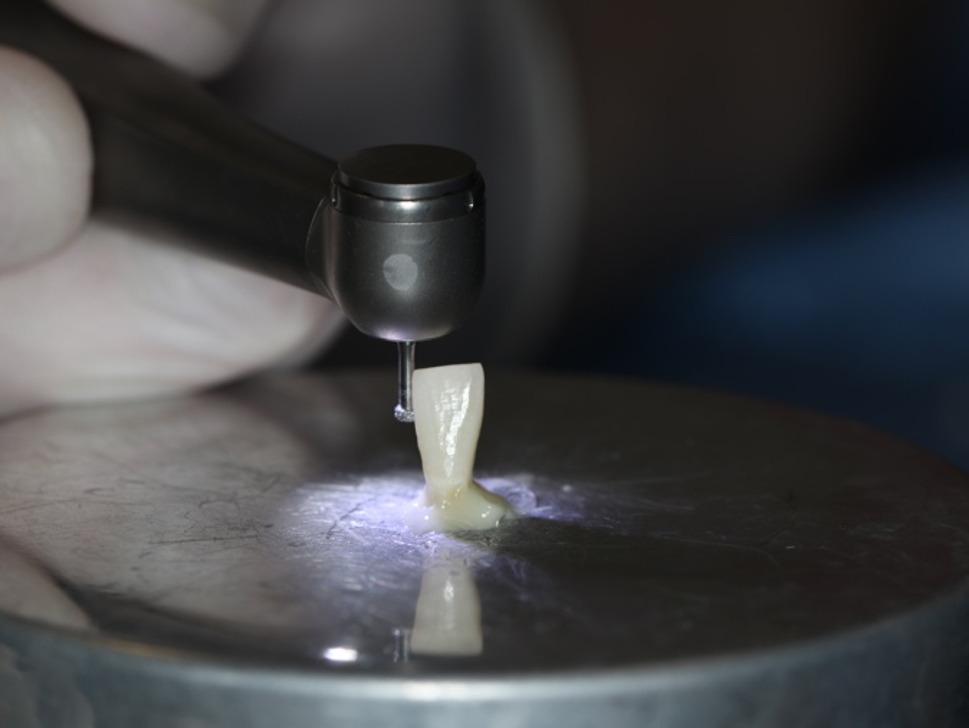
Marking with a bur with a penetration depth of 0.3 mm

Firstly, the IPR procedure was applied to the samples, which were prepared and placed on the pulpal microcirculation model (Fig. 4). To achieve clinical standardization of the pressure during the procedure, all IPR and polishing processes and measurements were carried out by a single calibrated operator (E.B.). The amount of IPR was determined to be 0.3 mm [38]. In order to standardize the amount of IPR, a mark was made on the lateral walls of the teeth with a 0.3 mm penetration depth and 1 mm long diamond bur (Intensiv, 818 102AC, Montagnola, Switzerland) (Figs. 5 and 6). The deepest point of the mark was dyed, and the IPR was applied until the dyed mark was erased from the mesial and distal surfaces of the samples.

**Fig. 7:**
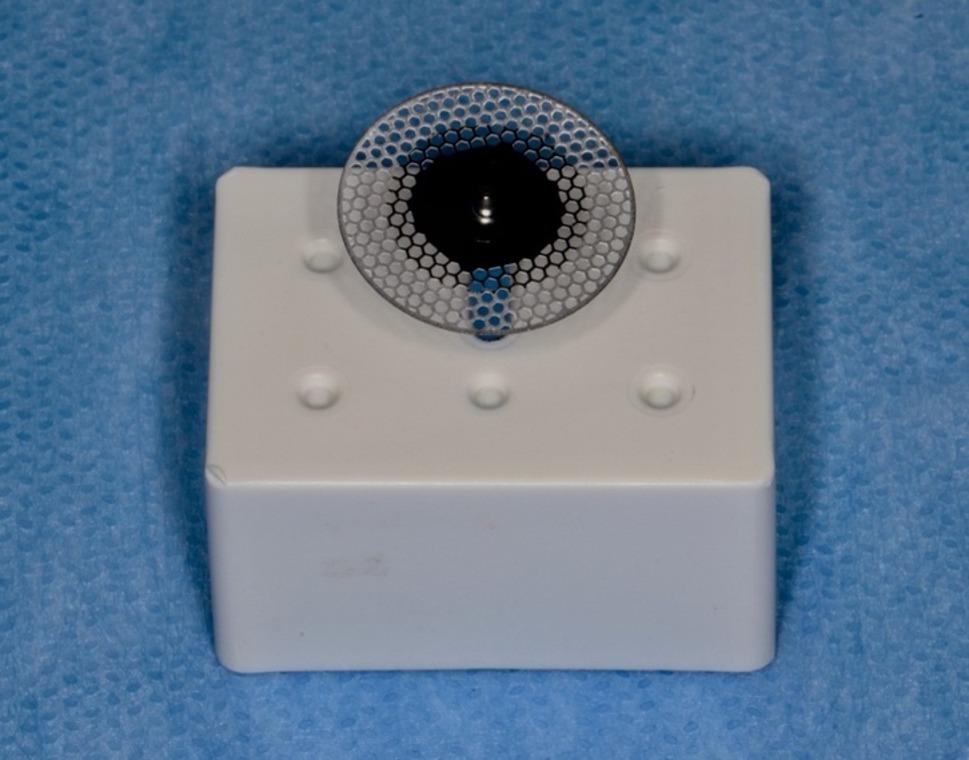
Perforated disk used in IPR procedure application (Komet, 8934 A.180, GmbH, Lemgo, Germany)

**Fig. 8:**
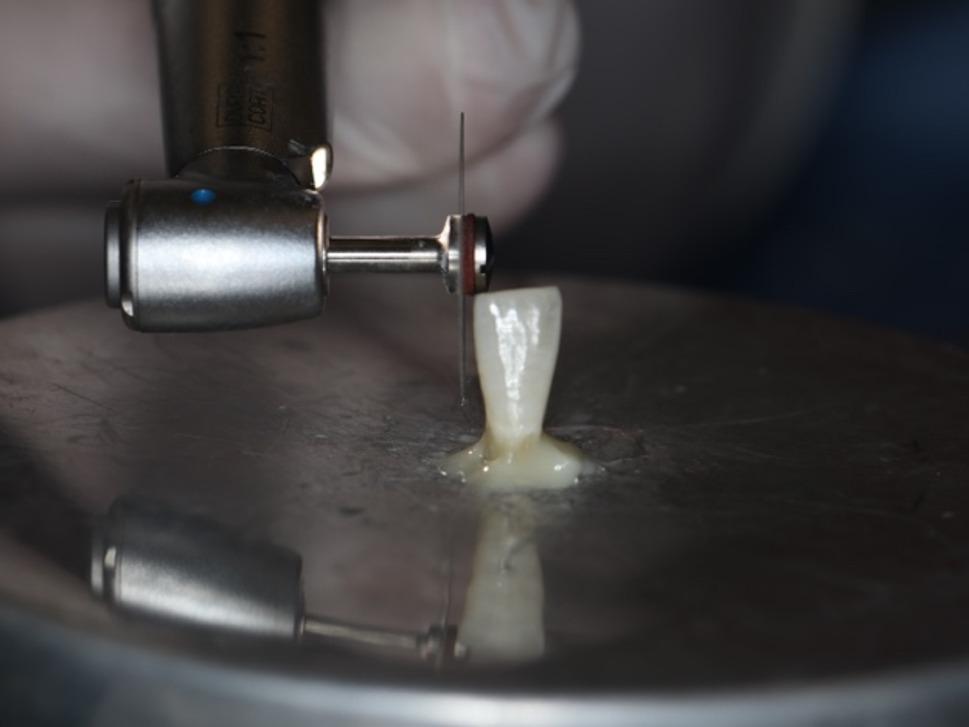
IPR application with perforated disc

In present study, a perforated disk (Komet, 8934 A.180, GmbH, Lemgo, Germany) with a diamond coating of 3 mm diameter on both surfaces, a thickness of 1.5 mm on the working part, and a diameter of 180 mm was used for the IPR (Fig. 7). IPR was applied at the recommended range rotation speed of 5000 rpm and dry according to the manufacturer’s instructions (Fig. 8) [12]. IPR was applied when the initial intrapulpal temperature (T_0_) of the teeth was within physiological limits. For each sample, the temperature changes that occurred during the process were recorded with a thermal camera, and the final temperature (T_1_) and the highest temperature (T_max_) were evaluated.

**Fig. 9:**
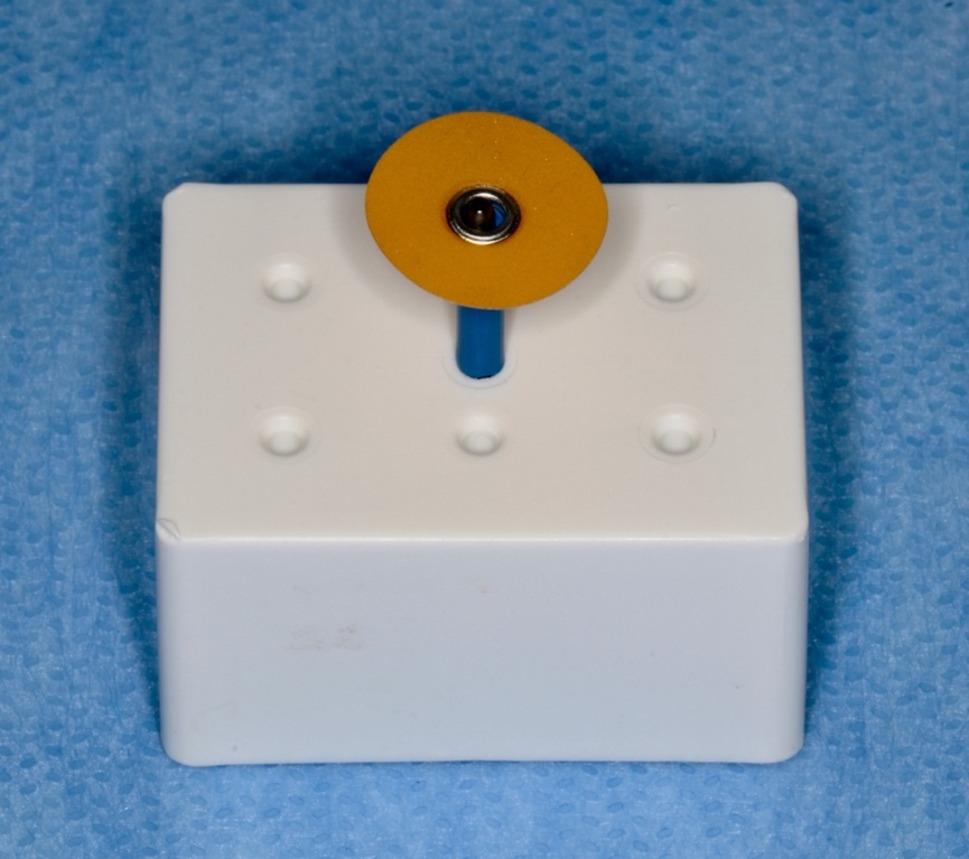
Polishing disc used during polishing (3 M ESPE, 8692 F, St Paul, Minn)

**Fig. 10:**
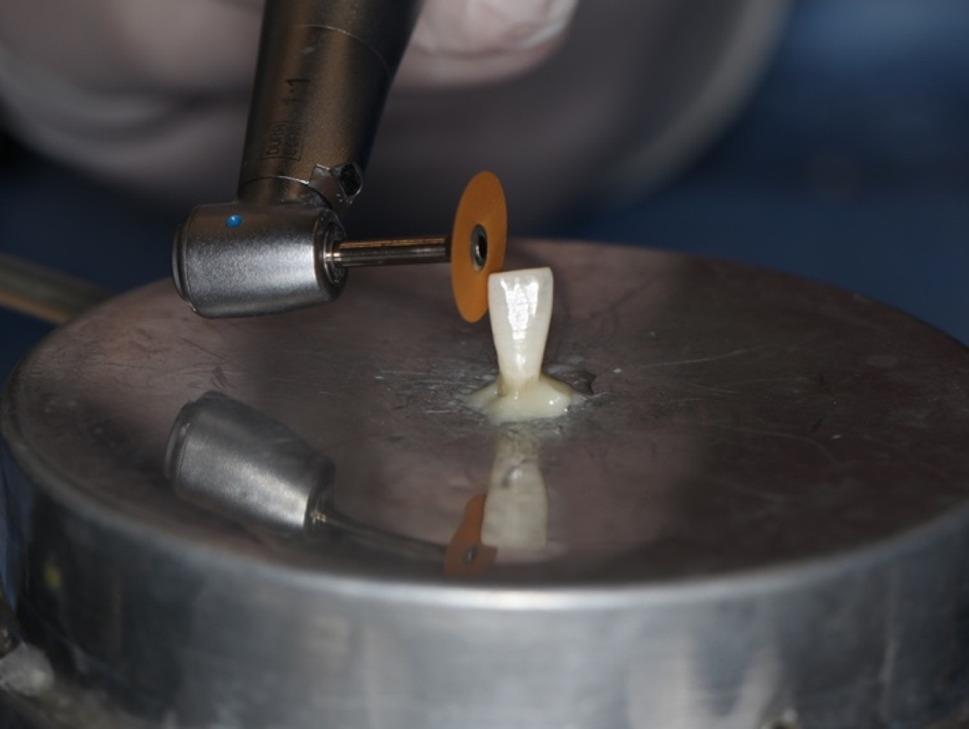
Polishing with Sof-Lex disc

As a second stage, as a reference to the studies in the literature which resulted with a smoother surface than the untreated enamel surface with the polishing after IPR, the polishing was applied for 80 seconds. [12, 28, 38] Sof-Lex polishing discs with fine grain size, 12.7 mm diameter aluminum oxide coated and flexible were used (3 M ESPE, 8692 F, St. Paul, Minn) (Fig. 9) [6, 28, 38]. The initial pulp chamber temperature (T_0_), 45th second (T_1_), 60th second (T_2_), and 80th second (T_3_) temperatures were recorded [12, 28, 38]. The polishing process was applied dry on one surface of the teeth according to the manufacturer’s instructions, and with water cooling on the other surface [28, 38]. The polishing disc was used with a speed of 5000 rpm according to the instructions and was changed for each application on each tooth (Fig. 10).

**Fig. 11:**
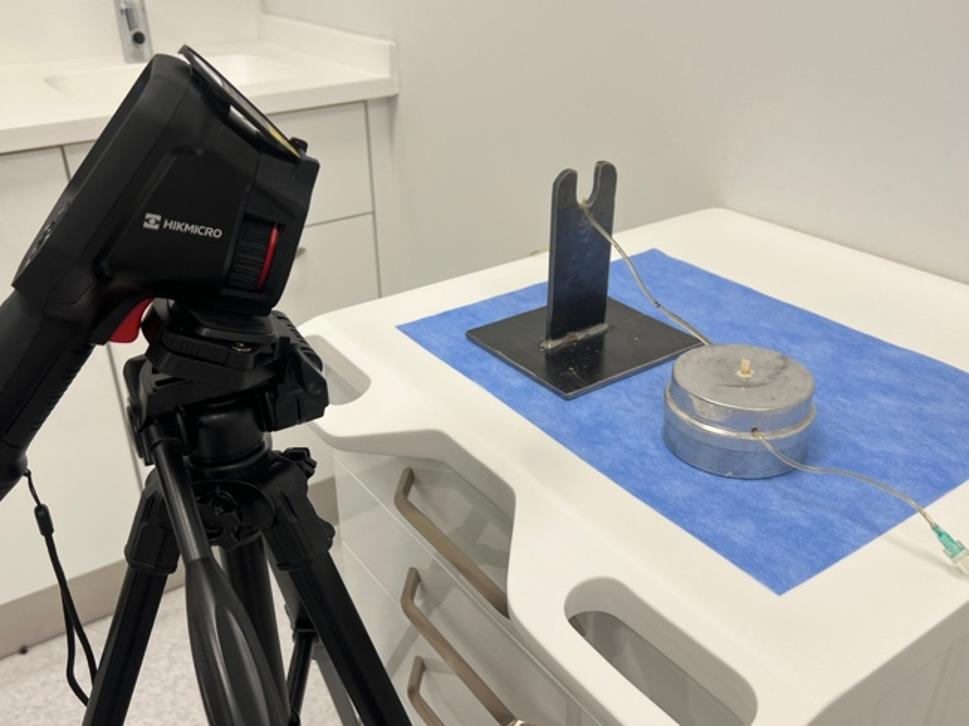
Thermal camera fixed 15 cm away from the experimental setup

**Fig. 12:**
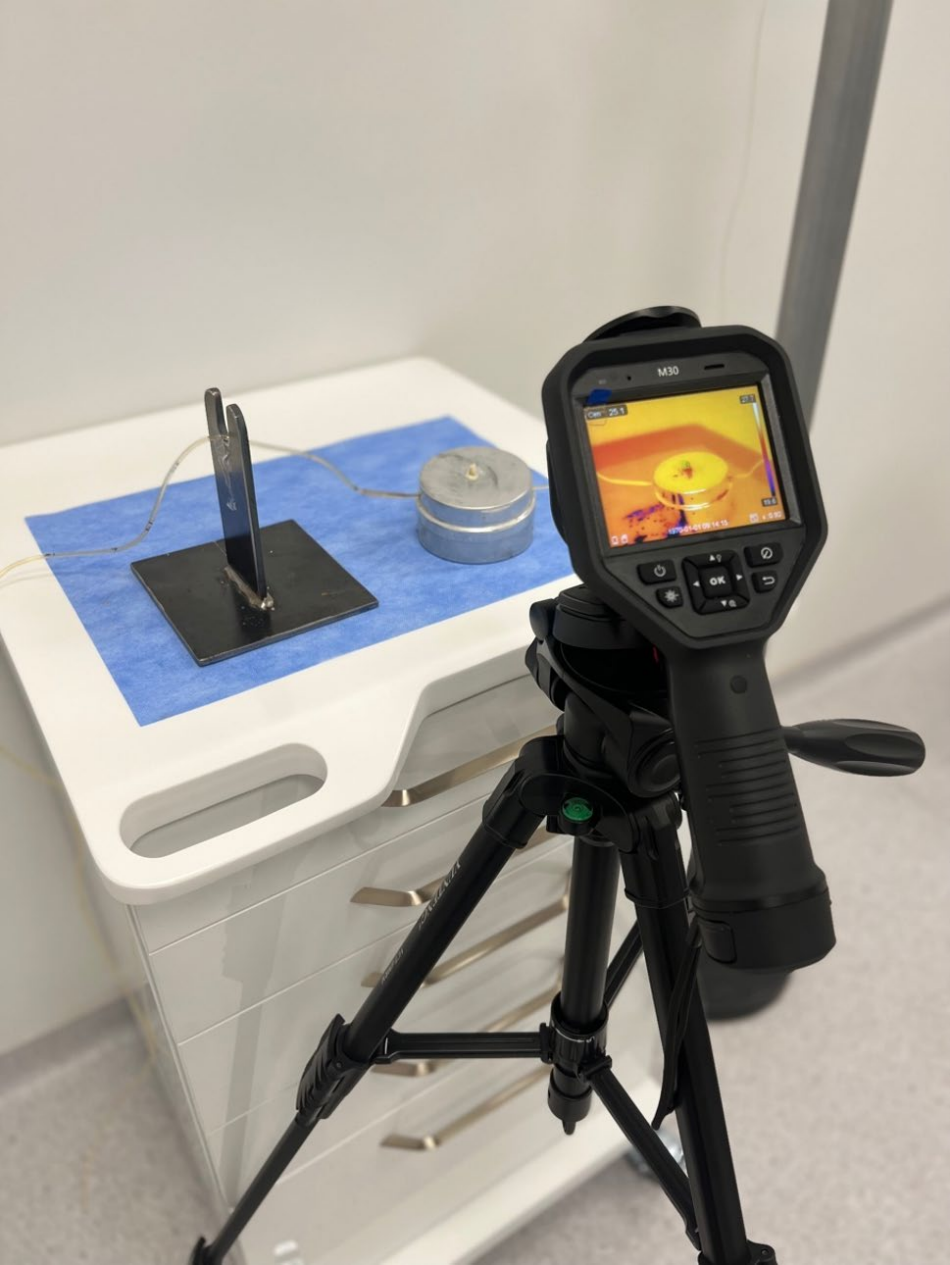
Recording the temperature change during IPR and polishing with a thermal camera

In present study, the M10 thermal camera, which can measure between − 20 °C and 550 °C, was used to record the temperature that occurred during the IPR and subsequent polishing (Hikmicro, M10, Hangzhou, Zhejiang). To standardize the recordings for all samples, the thermal camera was fixed at a distance of 15 cm from the microcirculation model (Fig. 11) [4]. The experiment was carried out at room temperature of 24 ± 1 °C, where environmental conditions were controlled. Temperature change and the highest temperature values during the IPR and polishing were recorded (Fig. 12). The temperature difference (ΔT) was determined by measuring the difference between the initial temperature and the highest temperature value. Positive ΔT indicates an increase in temperature in the pulp chamber, and negative ΔT indicates a decrease in temperature in the pulp chamber. The relationship between the temperature change and the critical value of 5.5 °C suggested by Zach and Cohen was evaluated [21]. 

While evaluating the findings obtained in our study, the SPSS (Statistical Package for the Social Sciences) 24.0 program was used for statistical analysis. In addition to descriptive statistical methods (Mean, Standard Deviation, Median, Frequency, Ratio, Minimum, Maximum), the Friedman Halton test was used to compare the changes over time, and the Wilcoxon Signed Rank test was used to determine the differences between measurements. The Mann-Whitney U test was used to evaluate the differences among groups. Significance was evaluated at *p* < 0.01 and *p* < 0.05 levels.

## Results

The initial temperature (T_0_), final temperature (T_1_), maximum temperature (T_max_) and the temperature change (ΔT) observed in the pulp chamber during the 0.3 mm IPR applied to each tooth are shown in Table 1. T_1_ and T_max_ were measured at the same temperature and varied between 21.8 °C and 30 °C. ΔT was measured as an average of 2.74 ± 1.4 °C. Only two samples showed a temperature change above the critical value of 5.5 °C.

**Table 1:** Temperature change average of measurements for IPR group

	Mean ± SD	Min-Max
*T*_0_ (ºC)	21.89 ± 0.68	20.3–23
*T*_*1*_ *(ºC)*	24.63 ± 1.84	21.8–30
*T*_*max*_ *(ºC)*	24.63 ± 1.84	21.8–30
*∆T (ºC)*	2.74 ± 1.4	0.9–7.3

*∆T* T_max_- T_0,_ *SD* Standart Deviation, *Min* Minimum, *Max* Maximum

A comparison of the polishing procedure applied to both surfaces of the teeth is shown in Table 2.

**Table 2:** Evaluation of temperature change measurements in water cooling and dry polishing groups

	Water cooling		Dry		
	Mean±SD	Min-Max	Mean±SD	Min-Max	^a^p
T_0_ (ºC)	21.74 ± 0.78	20.5–23.1	21.79 ± 0.85	20.5–23.1	0.830
T_1_ (ºC)	23.21 ± 1.03	21.7–24.9	24.09 ± 1.5	21.4–27	0.049*
T_2_ (ºC)	23.86 ± 1.01	22.2–25.5	24.99 ± 1.52	22.3–27.5	0.023*
T_3_ (ºC)	24.45 ± 1.08	22.7–26.3	25.52 ± 1.58	22.7–27.9	0.038*
T_max_ (ºC)	24.48 ± 1.07	22.7–26.3	25.52 ± 1.58	22.7–27.9	0.045*
∆T (ºC)	2.74 ± 0.44	1.8–3.5	3.73 ± 0.95	2.2–5.3	0.001**

*∆T* T_max_- T_0_, *SD* Standart Deviation, *Min* Minimum, *Max* Maximum

^a^Mann Whitney U Test

^**^p < 0.01, ^*^p < 0.05

The average value of the initial temperature (T_0_) measurements of the water cooling polishing group was 21.74 ± 0.78°C. At the dry polishing group, the average T_0_ was measured as 21.79 ± 0.85°C. T_0_ measurements of both groups do not show a statistically significant difference (0.830; *p* > 0.05).

The average temperature measurements at the 45^th^ second (T_1_) of the water cooling and dry polishing groups were determined as 23.21 ± 1.03°C and 24.09 ± 1.5°C, respectively. T_1_ measurements of both groups show a statistically significant difference (*p* = 0.049; *p* < 0.05).

The average 60^th^ second (T_2_) temperature measurements of the water cooling and dry polishing groups were determined as 23.86 ± 1.01°C and 24.99 ± 1.52°C, respectively. T_2_ measurements of both groups show a statistically significant difference (*p* = 0.023; *p* < 0.05).

The average temperature measurements at the 80^th^ second (T_3_) of the water cooling and dry polishing groups were determined as 24.45 ± 1.08°C and 25.52 ± 1.58°C, respectively. T_3_ measurements of both groups show a statistically significant difference (*p* = 0.038; *p* < 0.05).

The average of maximum (T_max_) temperature measurements for the water cooling and dry polishing groups were determined as 24.48 ± 1.07°C and 25.52 ± 1.58°C, respectively, and they showed a statistically significant difference (*p* = 0.045; *p* < 0.05). Different temperature values were detected between T_max_ and T_3_ measurements only in the two samples in the water cooling group. In all other samples, T_max_ and T_3_ were measured at the same value. T_1_, T_2_, T_3_, and T_max_ temperature measurements of the dry polishing group were found to be higher.

The average ΔT temperature measurements in the water cooling and dry polishing groups were measured as 2.74 ± 0.44°C and 3.73 ± 0.95°C, respectively. The temperature change measured in the water cooling polishing group was between 1.8 and 3.5°C, and in the dry polishing group, it was measured between 2.2 and 5.3°C. ΔT temperature measurements of the water cooling and dry polishing groups show statistically significant differences (*p* = 0.001; *p* < 0.01). The ΔT temperature measurement of the dry polishing group was found to be higher. However, no temperature increase above the critical value of 5.5°C was detected in both groups.

A comparison of measurements at the dry and water cooling polishing groups is presented in Table 3. The change in T_3_ temperature measurement compared to T_0_ measurement in the water cooling group was found to be statistically significant (*p* = 0.001; *p* < 0.01). According to the pairwise comparisons, a statistically significant difference was found between the T_0_ temperature measurement and the T_1_, T_2_, and T_3_ temperature measurements (*p* = 0.001; *p* < 0.01). The difference between the T_1_ temperature measurement and the T_2_ and T_3_ temperature measurements was found to be statistically significant (*p* = 0.001; *p* < 0.01). The difference between the T_2_ temperature measurement and the T_3_ temperature measurement was also found to be statistically significant (*p* < 0.001; *p* < 0.01). In the dry polishing group, the change in T_3_ temperature measurement compared to T_0_ measurement was found to be statistically significant (*p* = 0.001; *p* < 0.01).

**Table 3:** Comparison of measurements in water cooling and dry polishing groups

	Temperature measurments				
	T_0_	T_1_	T_2_	T_3_	
	Mean ± SD	Mean ± SD	Mean ± SD	Mean ± SD	^a﻿^p
Water cooling	21.74 ± 0.7	23.21 ± 1.0	23.86 ± 1.0	24.45 ± 1.1	0.001**
Dry	21.79 ± 0.8	24.09 ± 1.5	24.99 ± 1.5	25.52 ± 1.6	0.001**

*∆T* T_max_- T_0_, *SD* Standart Deviation, *Min* Minimum, *Max* Maximum

^a﻿^Friedman Halton test

^**^p < 0.01, ^*^p < 0.05

## Discussion

Polishing subsequent to IPR is an essential step that significantly reduces enamel surface roughness. [6, 7, 39] However, no study in the literature examines whether the heat generated during the polishing subsequent to IPR causes a temperature increase in the pulp chamber. There is no study has examined temperature changes during IPR using a microcirculation model. In this study, the temperature change in the pulp chamber during polishing, both with and without water cooling after IPR, was measured by simulating pulpal microcirculation with a thermal camera and the results were compared.

While the temperature change in the pulp chamber was examined in the studies, the role of vascular microcirculation of the pulp in thermal behavior has not been evaluated. However, in the presence of an external stimulus that transfers heat to the dentin-pulp complex, it has been argued that the vascular microcirculation of the pulp is one of the important factors that ensure intrapulpal temperature regulation [25]. Therefore, in studies where pulpal microcirculation is not simulated, it is possible to obtain higher and misleading values when evaluating the temperature change in the pulp chamber [25, 27]. 

In studies simulating pulpal microcirculation, the distilled water flow rate for simulating pulpal microcirculation was determined as 0.026 ml/min [26, 27, 32, 39]. Kodonas et al. adjusted the flow rate to 1 ml/min to prevent the flow from being interrupted [25]. In present study, the flow was adjusted to 0.026 ml/min with a flowmeter to obtain results closer to in vivo conditions. The distilled water flow in the pulpal microcirculation model was provided at room temperature in many studies [26, 35]. In some studies, distilled water flow was provided at 37 °C, arguing that it better reflects physiological conditions, but it was stated that there was a temperature loss from the water bath to the pulp chamber [25]. Since additional arrangements were needed in the model designed for pulpal microcirculation simulation to fix the temperature at 37 °C without any temperature loss, the water flow in present study was provided at room temperature.

Studies have examined the temperature increases with thermocouples during bonding [31], adhesive cleaning procedures after debonding [30, 32], and during IPR [4, 29, 40–42]. However, it has been stated that contact loss may occur with thermocouples, which measure from a single point, and open thermocouple junctions may cause errors in temperature measurement [43, 44]. The most important features of thermal cameras are their ability to measure without contacting the object, their high sensitivity, and fast response times [44]. Having their own analysis programs and the ability to examine the imaged object electronically are among their biggest advantages. The ability to make measurements over a large surface area and identify excessive hot spots are other positive aspects [43]. It has been argued that this non-invasive measurement method can be easily used under in vivo conditions [45]. Therefore, in present study, it is aimed to address the disadvantages of thermocouples by using a thermal camera to measure temperature changes during IPR, following the method of a previous study that utilized a thermal camera [5]. 

Studies have indicated that the most common area for applying IPR in orthodontics is the anterior of the lower arch [17, 18, 46]. Thus, it has been argued that the effect of IPR on the lower incisors have significant importance for orthodontic practice [40]. In the present study, we decided to use extracted human lower incisor teeth for orthodontic and periodontal reasons to obtain clinically consistent results.

Different materials have been used in studies evaluating temperature changes resulting from IPR, including metal abrasive strips [4, 29, 40–42], diamond-coated perforated discs [5, 7, 29, 40], tungsten carbide burs [5, 7], diamond burs [5, 29, 41, 42], and the Ortho-strip system [29, 42], followed by polishing with Sof-Lex discs of various particle sizes [6, 7, 9, 10, 16, 28, 38, 46–49]. Previous studies suggest that IPR with a diamond-coated perforated disk followed by polishing with fine-grained Sof-Lex discs is most successful in achieving a smooth surface [6, 38, 46]. In line with the results, fine-grained Sof-Lex polishing discs were used in present study.

The literature shows variations in the amount, duration, and rotation speed of the micromotor during IPR and polishing [4, 5, 7, 27, 38–41, 47]. In the present study, IPR was applied by creating 0.3 mm deep guide grooves and standardizing the procedure by using the disk at the lowest speed of 5000 rpm, as per the manufacturer’s instructions [50]. 

For the polishing procedure, different grain-sized discs and various methods such as strokes [7, 16, 47], duration [9, 12, 28, 38], rotation speed of the micromotor [12, 19, 38], and water cooling were considered [38]. The present study planned the polishing process to be applied for 80 s without water cooling on one surface and with water cooling at a speed of 5000 rpm on the other, aiming to evaluate the effect of water cooling on temperature increase.

When examining temperature changes during IPR, it was found that the temperature increase in the present study was higher than Baysal et al. [4] and Omer and Al Sanea’s studies [29]. However, the amount of IPR was less, but the temperature change was higher in present study, which might be due to the use of adult teeth and the measurement method (thermal camera vs. thermocouple) of the studies above. Although the teeth included in present study were not evaluated in terms of the age, they were evaluated in terms of pulp chamber width, whereas in studies it has been discussed that the decreased width of the pulp chamber due to the use of adult teeth causes the transmitted heat to be absorbed until it reaches the pulp chamber [4, 29, 43]. Only one study reported using a thermal camera, showing an average temperature increase of 7.3 °C, during 0.25 mm IPR with diamond-coated perforated disk under air cooling [5]. In the present study, despite creating deeper guide grooves, only two samples exhibited a temperature change above the critical threshold, likely due to the simulation of pulpal microcirculation, which aids in regulating temperature.

There is no studies in the literature have evaluated temperature changes during polishing subsequent to IPR. However, research on polishing composite and amalgam materials demonstrates a significant temperature increase with dry polishing compared to water cooling [22–24]. The findings of the present study align with these observations, as no significant increase in intrapulpal temperature was observed when water cooling was applied during polishing, consistent with reports from other materials. The temperature change in our study, 2.74 ± 0.44°C in the water cooling polishing group and 3.73 ± 0.95°C in the dry polishing group, was lower than those in these studies [22–24]. This may be due to the different conductivities of the filling materials used in the studies, which involved cavity preparation.

Like all in vitro studies, this study has certain limitations. Although the pulp chamber width was assessed using periapical radiographs, future studies can be designed by determining spesific age ranges of extracted teeth, creating different tooth groups, and increasing the sample size. Future research is also needed to evaluate various IPR materials and different particle sizes of polishing discs. Although the procedures were performed by a single operator to standardize clinical conditions as much as possible, variations in applied pressure may have occurred, which represents a limitation of the study. Since thermal camera measure on the tooth surface, whereas thermocouples measure from the pulp chamber, future studies could compare results obtained from both measurement methods. Although our study simulated pulpal microcirculation, it didn’t consider the influence of adjacent periodontal tissues on temperature rise. Nevertheless, the findings of this study may provide valuable insights for future in vivo studies on this topic.

## Conclusion

In conclusion, although a statistically significant temperature increase was observed during both IPR and polishing, only two samples showed a temperature rise above 5.5 °C during IPR. The higher temperature increase with dry polishing compared to water cooling suggests that water cooling is effective in controlling temperature rise. Importantly, no temperature increase above 5.5 °C occurred as a result of polishing with or without water cooling. The temperature increase recorded during the 80-second polishing with Sof-Lex discs following IPR was not sufficient enough to harm pulp tissue.

## Supplementary Information

Supplementary Material 1

## Data Availability

The datasets used and/or analysed during the current study are available from the corresponding author on reasonable request.
